# Feelings-As-Embodied Information: Studying the Role of Feelings As Images in Emotional Disorders

**DOI:** 10.3389/fpsyg.2018.00186

**Published:** 2018-02-20

**Authors:** Alexandru I. Tiba

**Affiliations:** ^1^Department of Psychology, University of Oradea, Oradea, Romania; ^2^Private Practice in Clinical Psychology, Oradea, Romania

**Keywords:** emotional disorders, embodied simulations, emotional reasoning, imagery of feelings, clinical cognition, feelings as embodied information

Our conscious experience of emotions (e.g., feeling happy or anxious) and thinking (e.g., judgements such as concluding that a situation is dangerous) have always been viewed in interaction. One way our feelings interact with our thinking is through judgments we make about external situations based on how we feel about them rather than on objective facts. We may conclude that we will get a salary raise because we feel good about the letter we received rather than on the facts that suggest the raise (Schwarz and Clore, 1988). Moreover, it has been demonstrated that when we provide anxious people with information about fear reactions and feelings in various situations, they interpret those situations as being more dangerous than non-anxious people do (emotional reasoning, e.g., *Thinking the roof will collapse because you feel anxious*). Exaggerated use of negative feelings as source for judging danger (i.e., emotional reasoning) has been demonstrated to have clinical relevance both as maintenance factor (Arntz et al., 1995) and as a treatment target in cognitive therapy for emotional disorders (Beck, 1976; Beck and Haigh, 2014). Therefore, clarifying the processes involved in using feelings as information for reasoning may be important both for the understanding of the mechanisms involved and for the treatment of emotional disorders.

The dominant view of the representation of feelings in our mind involves an amodal account of representation (e.g., Lang, 1979; Bower, 1981). The amodal account of the cognitive representation of feelings suggests that after we experience emotional states, in our cognitive system, they are transformed into a different informational format (semantic structures independent of the qualities of the emotional states and emotional systems from which they originate). They are represented in language-like format (propositions about responses such as *I feel anxious*; *I blush*) which serves as input for further thinking (Wyer et al., 1999). In other words, after experiencing feelings, in our mind, they are dis-embodied information (feelings-as-dis-embodied information, FaDI). As such, providing information about feelings has an effect on thinking that emerges from their dis-embodied informational properties, independent from other factors such as their composition or the affective systems that produced them (Wyer et al., 1999).

In the last decades, however, a different idea has been promoted in cognitive science. In certain contexts, the representation of feelings in our mind may also be embodied (as partial re-creations of the neural and body pattern that was active during the emotional experience, Niedenthal, 2007). Thus, feelings may have offline interactions with other processes (judgements) as embodied information. This idea has important clinical implications for our understanding of the emotional reasoning process and of the mechanisms of emotional disturbances. In the present article, I focus on the relationship between feelings and thinking in clinical context from the perspective of embodied cognition (Niedenthal, 2007).

## Feelings as embodied simulations

The embodied views of emotional cognition suggest that emotional states do not disappear after they are experienced. There is increasing empirical support (Vermeulen et al., 2007; Niedenthal et al., 2009; Oosterwijk et al., 2010; Davis et al., 2015, 2017) and theoretical advances (Niedenthal et al., 2005; Niedenthal, 2007; Barsalou, 2009; Barrett et al., 2014) for the fact that emotional states can be co-opted as embodied simulations of feelings (partial re-creation of emotional states) in emotional cognition (feelings-as-embodied information, FaEI). Once we have the experience of feelings, these experiences are captured in memory (in the same affective format as they were experienced) being accessible for later retrieval. Whilst usually they function unconsciously, when they are deliberate and conscious, they may appear as imagery (Barsalou, 1999, 2009; Niedenthal, 2007). When we imagine an apple we usually re-create (mentally simulate) the visual perception of an apple (Barsalou, 1999). Similarly, when we imagine “feelings of happiness” we mentally simulate the experience of feeling happy (along with other components of the situated conceptualization of feeling happy, Barrett et al., 2014). From this theoretical position, feelings can be types of perception (online cognition, Barrett and Bar, 2009), or images (as offline cognition, Barsalou, 2009). As such, feelings may also influence judgements via the properties of the simulations of emotional experiences (similar to higher impact of vivid rather than fade visual images of judgement). Moreover, the embodiment process also recruits in the simulation the neural systems involved in the emotional experience. Thus, the simulations of feelings may also reflect the properties of the affective systems implementing them (e.g., hyper-reactivity) in the interaction between emotional cognition and other types of reasoning (Niedenthal, 2007). It is important to mention that FaEI does not refute propositional representations in emotional imagery. It recognizes the existence of propositional representations but adds modal representations (Barsalou, 2009) thus avoiding an “imagery debate” problem (Pearson and Kosslyn, 2015).

Thus, the embodied cognition position on feelings brings two new implications which I suggest are important for the interaction between feelings and judgements in the clinical domain:

1. Off-line feelings may take the form of mental images (Barsalou, 2009). As mental images they have distinct characteristics (e.g., vividness that depends on the accessibility of affective systems) which may influence the interaction of feelings with judgements and may contribute to emotional disorders. Amodal theories of emotional imagery (e.g., Lang, 1979) reject the idea that feelings can be imagined based on affective format. Accordingly, the activation of the image of an emotional situation (involving propositions about stimuli, reactions) plays the role of “as if real” templates that trigger emotional reactions and feelings (Ji et al., 2017). From the perspective of amodal theories, feelings are not part of imagery. They are reactions to imagery.
2. The embodied simulation of feelings re-uses the neural processes involved in the simulated emotional experience (Niedenthal, 2007). By this mechanism, the differences in the affective systems re-used for simulation (e.g., hyper-reactivity in anxiety) may be reflected in the differences in the characteristics of the simulations (vividness, ability to elicit emotion) and their impact on emotion.

In the following section, I explore the idea that FaEI may be an important process involved in emotional reasoning and clinical phenomena. Furthermore, based on these implications of embodied perspective on feelings I point to several implications for psychological research and treatment.

## Implications of feelings-as-embodied information for emotional reasoning

In their seminal experiment on emotional reasoning, Arntz et al. (1995) asked participants with anxiety disorders and a healthy control group to read scripts about dangerous and safe situations (e.g., being in an elevator). In some scenarios, they also described anxious feelings (“*Suddenly you become very anxious*” p. 919). In other scenarios, only objective danger information was presented. After being presented scenarios that included information about feelings, participants judged the probability of danger as higher than after scenarios without information about feelings. Arntz and colleagues also asked participants to “*evaluate these events as if they are happening to you.…try to identify yourself with the description as much as possible*” (Arntz et al., 1995, p. 920). Evaluation of the situations *as if they are happening to you* most likely requires mental simulation of the events and of threatening information. Thus, the differences in the evaluation of danger between anxious and non-anxious participants may also be explained by the differences in the simulation of information about feelings.

Anxious patients may perceive more danger because they assess the danger based on highly vivid images of anxious feelings. Thus, the relation between feelings and thinking may be based also on a simulation heuristic, as in the case of the relation between mental imagery and judgment (Kahneman and Tversky, 1982; Schwarz, 2012). Such differences in the easiness/vividness of images of affect may depend on the reactivity of affective systems recruited in the simulation (similarly to the vividness of visual imagery that depends on the top-down connectivity to early visual cortex, Dijkstra et al., 2017). Anxious individuals are characterized by hyper-reactivity of affective systems (Etkin and Wager, 2007). Hence, they may simulate more vividly feelings of anxiety which may be used to assess danger as more probable. Consequently, the differences in judgments reflect more than favoring an emotional reasoning thinking “style.” They may reflect also the differences in the accessibility of simulations of anxious feelings related to hyper-reactivity of affective systems. Depressed individuals have difficulties in the simulation process and may not show increases in emotional reasoning (Berle and Moulds, 2013).

## Finding feelings-as-embodied information into clinical phenomena

Should the feelings function in relation to higher thinking also as embodied information, properties of FaEI such as feelings as imagery will be observable in clinical phenomena involving higher emotional cognition. For instance, if FaEI is important for clinical phenomena we can identify feelings as mental simulations (images of feelings). Furthermore, if simulations of feelings are important for the effect of feelings on higher cognition, then the properties resulting from FaEI (such as qualities of imagery of feelings) will both be recognizable and necessary to see such an effect.

## Affective forecasting

Affective forecasting refers to estimations of how one will feel in the future (Wilson and Gilbert, 2005). This concept was shown to be clinically relevant especially for depressed individuals who were shown to estimate less positive emotions than non-depressed individuals. Most studies measure affective forecasts by asking participants to estimate how they will feel. Yet, a recent study asked people to imagine how they will feel measuring affective simulations rather than cognitive processing affect (Marroquín and Nolen-Hoeksema, 2015). They found that affective forecasts in the form of simulations for future positive emotions (FaEI) are attenuated in individuals with dysphoria (Marroquín and Nolen-Hoeksema, 2015). These results show that distortions in FaEI such as imagining positive emotions may play an important role in disturbances of affect such as in non-clinical dysphoria.

## Imagining feelings

Similar to other studies investigating emotion in depressed individuals, Greden and his collaborators asked depressed individuals to imagine feeling positive or sad emotions while measuring facial electromyography responses (EMG). The results showed that people who were depressed and had higher pre-treatment EMG zygomatic values when they imagined positive feelings respond better to antidepressant treatment (Greden et al., 1984). As the EMG pattern in response to imagining emotions is a marker of emotional simulations (Oosterwijk et al., 2010), these results suggest that individual differences in the qualities of simulation of emotions are clinically important.

## Research and treatment implications

The proposal that feelings may act as embodied mental simulations suggests several directions for clinical research. First, would be to examine whether there are significant differences in the qualities of imagining feelings (e.g., vividness) in emotional disorders. Analogous to measures of vividness of kinaesthetic imagery (in which participants are asked to rate the clarity of imagining the sensation of movement), studies may measure the vividness of imagining feelings by asking participants to rate the clarity of imagining emotional states.

In line with studies that have investigated the imagery of emotional events (Holmes et al., 2016), researchers may examine whether the vividness of imagining positive feelings is attenuated in depressed patients. Similarly to findings of mental imagery of aversive events in anxious patients (Hirsch and Holmes, 2007), the vividness of imagining anxious feelings may be increased in anxious patients. Moreover, studies should clarify if vividness of imagining feelings moderates (or mediates) the emotional effects of factors involved in psychopathology (general mental imagery of emotional events, affective forecast, or emotional reasoning). Second, would be to explore the differences between clinical and non-clinical groups in imagining feelings according to the computational phases of imagery (generation, maintenance, transformation, and inspection, Pearson et al., 2013). For instance, depressed individuals may have difficulties in maintaining over time rather than generating images of positive feelings. Some studies suggest that such a deficiency in sustaining positive affect over time may be linked with anhedonia (Heller et al., 2009). If proven as a mechanism for emotional disorders, it could suggest new directions for treatment development. For instance, researchers may test the effect of new types of affective computerized training, in which depressed patients are required to repeatedly generate vivid images of positive affect. Researching the process of imagining specific feelings may conclude in developing theories referring to the mechanism of emotional disorders or specific treatment.

## Author contributions

The author confirms being the sole contributor of this work and approved it for publication.

### Conflict of interest statement

The author declares that the research was conducted in the absence of any commercial or financial relationships that could be construed as a potential conflict of interest.
